# Recombination in Vaccine and Circulating Strains of Porcine Reproductive and Respiratory Syndrome Viruses

**DOI:** 10.3201/eid1512.090390

**Published:** 2009-12

**Authors:** Bin Li, Liurong Fang, Zuofei Xu, Suyan Liu, Jianfeng Gao, Yunbo Jiang, Huanchun Chen, Shaobo Xiao

**Affiliations:** Huazhong Agricultural University, Wuhan, People’s Republic of China

**Keywords:** Porcine reproductive and respiratory syndrome virus, natural recombination, pathogenicity, China, viruses, dispatch

## Abstract

Em2007, a porcine reproductive and respiratory syndrome virus (PRRSV) variant with a unique 68 aa deletion in Nsp2, was recently isolated in China. Phylogenetic and molecular evolutionary analyses indicated that Em2007 is a natural recombinant between a vaccine strain of PRRSV and circulating virus. We also tested its pathogenicity in piglets.

Porcine reproductive and respiratory syndrome (PRRS) is now considered one of the most economically important diseases in countries with intensive swine industries. The causative agent, PRRS virus (PRRSV), is a member of the family *Arteriviridae* (*1*). The genome of PRRSV is ≈15 kb and encodes 9 open reading frames (ORFs). Two distinct genotypes of PRRSV share only ≈60% nucleotide identity and are represented by the North American prototype VR-2332 and the European prototype Lelystad virus (LV) (*2*). Sequence differences have also been found among isolates of the same genotype, particularly in the Nsp2 regions within ORF1a, and ORF5 (*3*). Mutation and genetic recombination play an important role in the evolution of PRRSV (*4**–**6*).

Since May 2006, porcine high fever syndrome, caused by highly pathogenic PRRSV and characterized by high fever and high death rates in pigs of all ages, has emerged in China and affected >20 million pigs (*7**–**9*). Genomic analysis showed that nearly all of the emerging highly pathogenic PRRSVs isolated from this outbreak share a unique discontinuous deletion of 30 aa in Nsp2 (*7**–**10*). However, a novel PRRSV variant, with a 68 aa deletion in Nsp2, emerged in central China in 2007. We report the unique genetic characteristics of this novel variant and its pathogenicity in piglets.

## The Study

At the end of 2007, a smaller cDNA fragment than the expected size was observed from a fetal piglet when a diagnostic reverse transcription–PCR (RT-PCR) was performed to amplify the unique genetic marker of the highly pathogenic PRRSV, indicating that a novel PRRSV variant was found. This strain, designated Em2007, was subsequently isolated and the full-length genomic sequence was determined. The genome of Em2007 was 15,272 bp, including the poly(A) tail (GenBank accession no. EU262603), and shared 87.6% and 57.9% sequence identity with VR-2332 and LV, respectively, indicating that Em2007 belongs to the North American genotype. The Nsp2 gene of Em2007 was 2,736 bp and encoded 912 aa, with a unique continuous deletion of 68 aa at positions 499–566, relative to strain VR-2332 (Technical Appendix). This unique deletion is substantially different from previous PRRSV isolates with deletions in Nsp2 (*3**,**7**–**11*).

To establish the genetic relationships of Em2007, we constructed phylogenetic trees using the neighbor-joining method based on the full-length genome. Results showed that Em2007 formed a minor branch, which was located in the middle of 2 clusters represented by CH-1a (the first PRRSV isolated in China in 1996) and JXA1 (the highly pathogenic PRRSV isolated in China in 2006), respectively (data not shown).

We also compared the sequence identity of individual Em2007 ORFs with representative PRRSV isolates and found that all ORFs have highest identity (>92%) with CH-1R (an attenuated vaccine strain used in China), except for Nsp2 (80.2%). Because recombinations have been reported in PRRSV in previous studies (*6*), we speculated that Em2007 is a mosaic. To test our hypothesis, we used 3 approaches to detect possible recombination events within Em2007.

**Figure 1:**
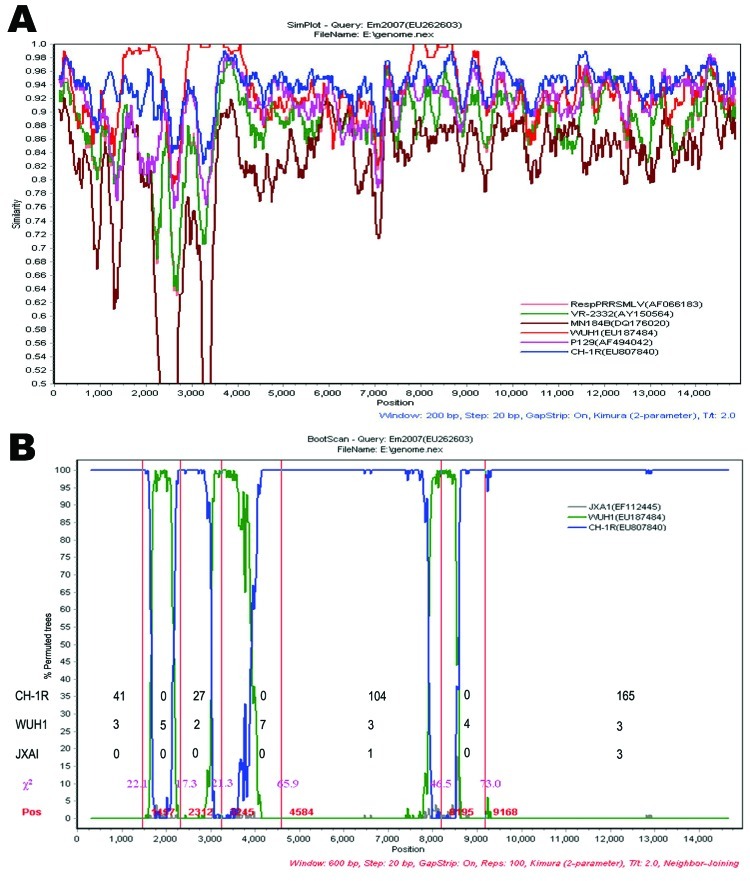
Recombination event analyses of the Em2007 strain of porcine reproductive and respiratory syndrome virus (PRRSV). A) Similarity plot analysis using Em2007 as query sequence. Analysis made use of a sliding window of 200 bases and a step size of 20 bases. The y-axis shows the percentage similarity between the selected PRRSV sequences and the query sequence. The other comparisons are not shown for clarity. B) Bootscan analysis using Em2007 as the query sequence. JXA1 is used as the outgroup to determine the breakpoints. The y-axis shows the percentage of permutated trees using a sliding window of 600 bases and a step size of 20 bases. Red vertical lines and numbers indicate the recombination breakpoints identified by the Genetic Algorithm for Recombination Detection (GARD). Pink numbers indicate the maximal χ^2^ value of each breakpoint. Numbers corresponding to CH-1R, WUH1, and JXA1 indicate the quantity of informative sites in 7 zones defined by 6 recombination breakpoints, respectively.

First, SimPlot, which calculates and plots the percent identity of a query sequence against a panel of reference sequences in sliding windows (*12*), was performed using Em2007 as a query. Based on a set of complete genome sequences, including 56 Chinese PRRSVs isolated during 1996-2008, three representative North American strains (VR-2332, MN184B, and P129), and 2 attenuated vaccine strains (RespPRRS, CH-1R) (the origin of all strains is listed in the Table), the SimPlot graph clearly showed that Em2007 was generally closer to CH-1R than to any other strain. However, there were 3 narrow zones showing disproportionately low levels of similarity between the 2 strains compared to other regions (Figure 1, panel A). Notably, the 3 narrow zones of Em2007 had high levels of similarity with WUH1 (a highly pathogenic PRRSV, isolated in Wuhan, China in 2006). These results indicated that Em2007 is a possible recombinant and CH-1R and WUH1 are 2 putative parental-like strains. Recombination was further analyzed by Bootscan, a program for the detection of recombination events, and the Genetic Algorithm for Recombination Detection (GARD) (*13*). Six potential recombination breakpoints, with maximal χ^2^, were found (Figure 1, panel B), indicating that 3 recombination events have taken place within Em2007. Two recombination fragments (1,457–2,312 and 3,245–4,584) are located in Nsp2; the third (8,195–9,168) is located in Nsp9.

**Table:** Origin and GenBank accession numbers of 61 PRRSV isolates from China and representative strains from North America used in this study*

Isolate no.	Strain	Country of origin	GenBank accession no.		Isolate no.	Strain	Country of origin	GenBank accession no.
1	CH-1a	China	AY032626		32	HUN4	China	EF635006
2	BJ-4	China	AF331831		33	HuN	China	EF517962
3	PRRSV01	China	FJ175687		34	JXwn06	China	EF641008
4	PRRSV02	China	FJ175688		35	07QN	China	FJ394029
5	PRRSV03	China	FJ175689		36	GD	China	EU825724
6	HB-1(sh)/2002	China	AY150312		37	CG	China	EU864231
7	HB-1-3.9	China	EU360130		38	NM1	China	EU860249
8	HB-2(sh)/2002	China	AY262352		39	07NM	China	FJ393456
9	GS2002	China	EU880443		40	XH-GD	China	EU624117
10	GS2003	China	EU880442		41	Em2007	China	EU262603
11	GS2004	China	EU880441		42	Henan-1	China	EU200962
12	CH2002	China	EU880438		43	Jiangxi-3	China	EU200961
13	CH2003	China	EU880440		44	SX2007	China	EU880434
14	CH2004	China	EU880439		45	WUH1	China	EU187484
15	HN1	China	AY457635		46	LN	China	EU109502
16	NB/04	China	FJ536165		47	SHH	China	EU106888
17	SHB	China	EU864232		48	GD2007	China	EU880433
18	CC-1	China	EF152486		49	BJ	China	EU825723
19	HUB1	China	EF075945		50	07BJ	China	FJ393459
20	HUB2	China	EF112446		51	HN2007	China	EU880437
21	HEB1	China	EF112447		52	07HEBTJ	China	FJ393458
22	JSyx	China	EU939312		53	07HEN	China	FJ393457
23	JX143	China	EU708723		54	CH-1R	China	EU807840
24	JXA1	China	EF112445		55	GS2008	China	EU880431
25	SY0608	China	EU144079		56	XL2008	China	EU880436
26	TP	China	EU864233		57	HPBEDV	China	EU236259
27	JX2006	China	EU880432		58	VR-2332	USA	AY150564
28	S1	China	DQ459471		59	P129	USA	AF494042
29	BJsy06	China	EU097707		60	RespPRRSMLV	USA	AF066183
30	TJ	China	EU860248		61	MN184B	USA	DQ176020
31	NX06	China	EU097706					

*PRRSV, porcine reproductive and respiratory syndrome virus.

Phylogenetic trees of nucleotide sequences of each recombination region defined by GARD, including flanking regions, were further reconstructed by the neighbor-joining method. A large discrepancy (p<0.001, by Shimodaira-Hasegawa test) between phylogenetic trees inferred for each recombination region constitutes powerful evidence for recombination (Technical Appendix). In addition, a retrospective survey found that the fetal piglet from which Em2007 was isolated was from a farm in Wuhan, China, and CH-1R was used on this farm to control PRRS, indicating the potential for recombination between CH-1R and WUH1. This evidence further supported the possibility that Em2007 is a natural recombinant between CH-1R and WUH1.

To test the virulence of Em2007, 40-day-old PRRSV-free piglets (9 piglets in each group) were inoculated intramuscularly with 10^5.0^ mean tissue cultures infectious doses/2 mL of Em2007, CH-1a, and WUH1, respectively. Control piglets were inoculated with Dulbecco minimal essential medium. Clinical signs and rectal temperature were recorded daily. Two piglets from each group were euthanized and necropsied at 7 and 10 days postinoculation, and organs including lung, brain, spleen, kidney, liver, intestines, and lymph nodes were collected for viral load analyses and histopathologic examinations. The remaining 5 piglets in each group were observed for 21 days to evaluate death rates.

**Figure 2:**
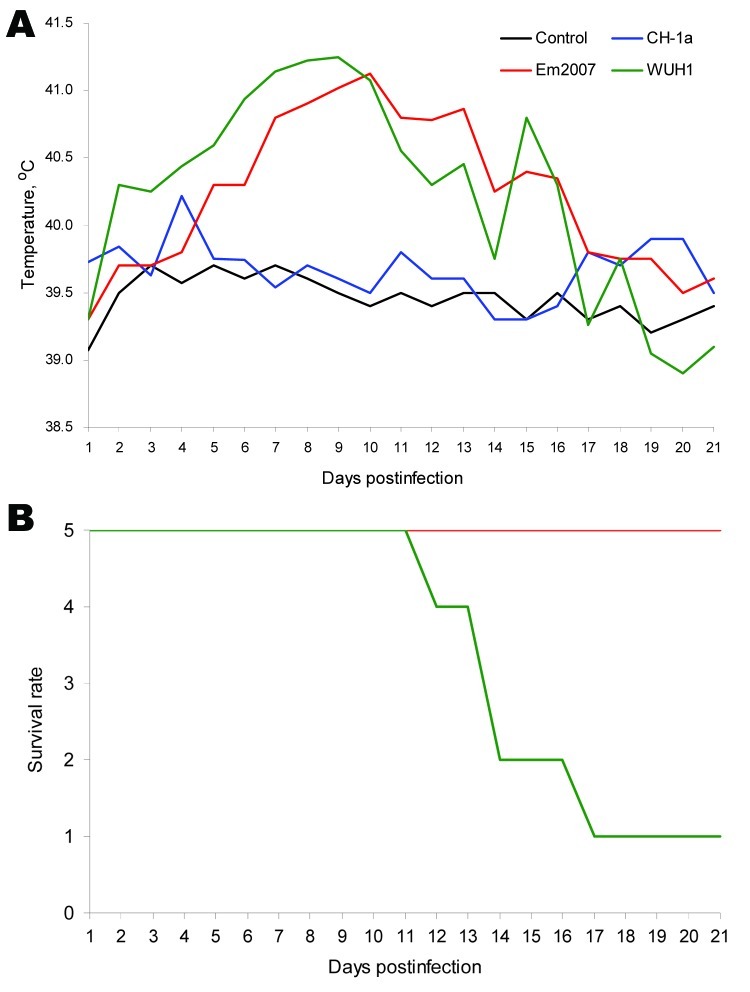
Pathogenicity comparison among the Em2007, CH-1a, and WUH1 strains of porcine reproductive and respiratory syndrome virus (PRRSV). Forty-day-old piglets (9 piglets in each group) free of PRRSV were inoculated intramuscularly with 10^5.0^ mean tissue culture infectious doses/2 mL of Em2007, CH-1a, WUH1, respectively. Two piglets from each group were euthanized and necropsied at 7 and 10 days postinoculation (dpi) for viral load analyses and histopathologic examinations. The remaining 5 piglets in each group were used to evaluate rate of death. Mean rectal temperature (A) and survival rate (B) of each group were recorded for 21 dpi.

Results showed that piglets inoculated with CH-1a experienced only temporary fever and mild respiratory symptoms. Obvious clinical signs, including inappetence, lethargy, high and continuous fever, red discolorations in the body, and blue ears were observed in piglets inoculated with WUH1 (Figure 2, panel A). Furthermore, severe interstitial pneumonia (Technical Appendix) and nonsuppurative encephalitis cases were also observed 7 and 10 days postinoculation (Figure 2, panel B). Four of 5 piglets died within 21 days after inoculation. Piglets inoculated with Em2007 also showed similar clinical signs to those seen in the WUH1 group. However, the interstitial pneumonia and nonsuppurative encephalitis were mild and no deaths occurred throughout the experimental period in Em2007 group. The results of viremia and viral load also indicated that Em2007 was more mild than WUH1, but of substantially higher virulence than CH-1a (data not shown).

## Conclusions

Em2007, a PRRSV variant with a unique continuous deletion of 68 aa in Nsp2, was isolated in China. This variant is a natural recombinant between an attenuated PRRSV vaccine strain CH-1R and a highly pathogenic PRRSV strain, WUH1. Animal experiments demonstrated that while Em2007 has higher virulence than CH-1a, the parental strain of CH-1R, it is attenuated relative to WUH1.

Previous studies have shown that genetic recombination occurs between attenuated vaccine strains of PRRSV grown together in culture (*14*). This study demonstrates for the first time that natural recombination can occur between vaccine and field strains, suggesting that live vaccines have the capacity to shape PRRSV evolution by homologous recombination with circulating virus.

## Supplementary Material

Technical Appendix Recombination in Vaccine and Circulating Strains of Porcine Reproductive and Respiratory Syndrome Viruses
