# Supplementary material for: Recombination in Vaccine and Circulating Strains of Porcine Reproductive and Respiratory Syndrome Viruses
Source: Emerg Infect Dis. 2009 Dec;15(12):2032–5. doi: 10.3201/eid1512.090390 (PMC3044526; doi:10.3201/eid1512.090390)
Supplement: Technical Appendix — Recombination in Vaccine and Circulating Strains of Porcine Reproductive and Respiratory Syndrome Viruses [file 09-0390_Techapp-s1.pdf]

Recombination in Vaccine and Circulating Strains of Porcine Reproductive and Respiratory Syndrome Viruses

Technical Appendix

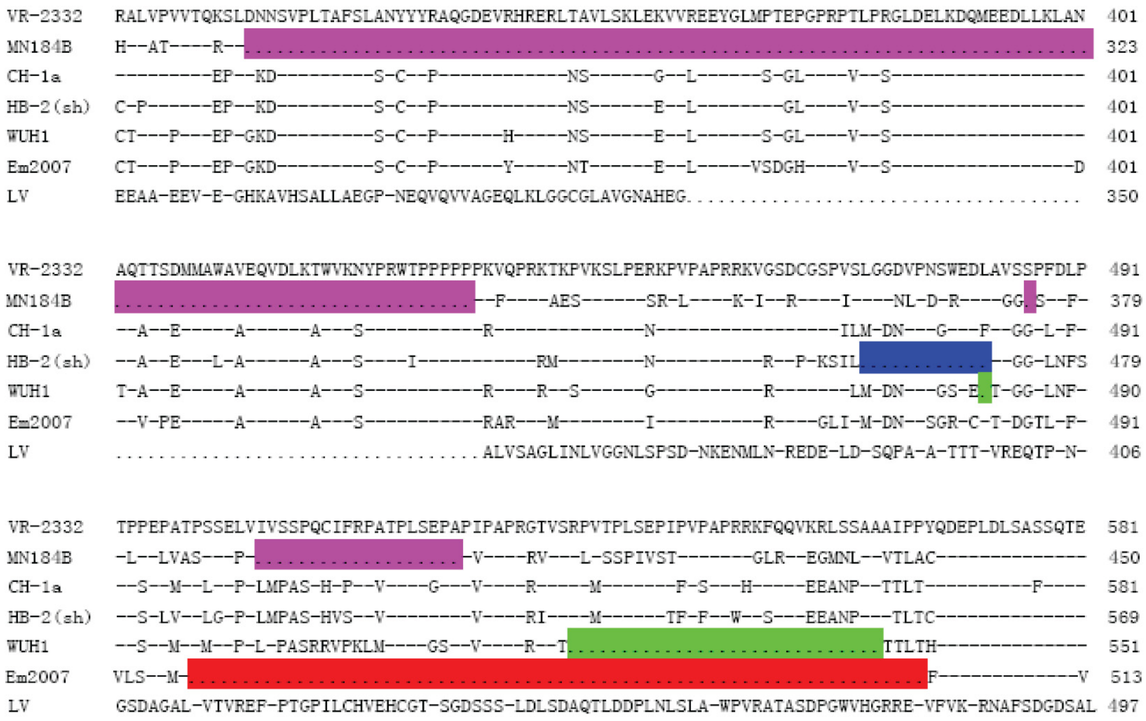

Figure 1. Genetic characteristics of Em2007 strain of porcine reproductive and respiratory syndrome virus (PRRSV) showing amino acid alignment of the Nsp2 deletion region of Em2007 with some representative PRRSV isolates. Conserved residues are indicated by dashes and the deleted amino acids are indicated by dots and color.

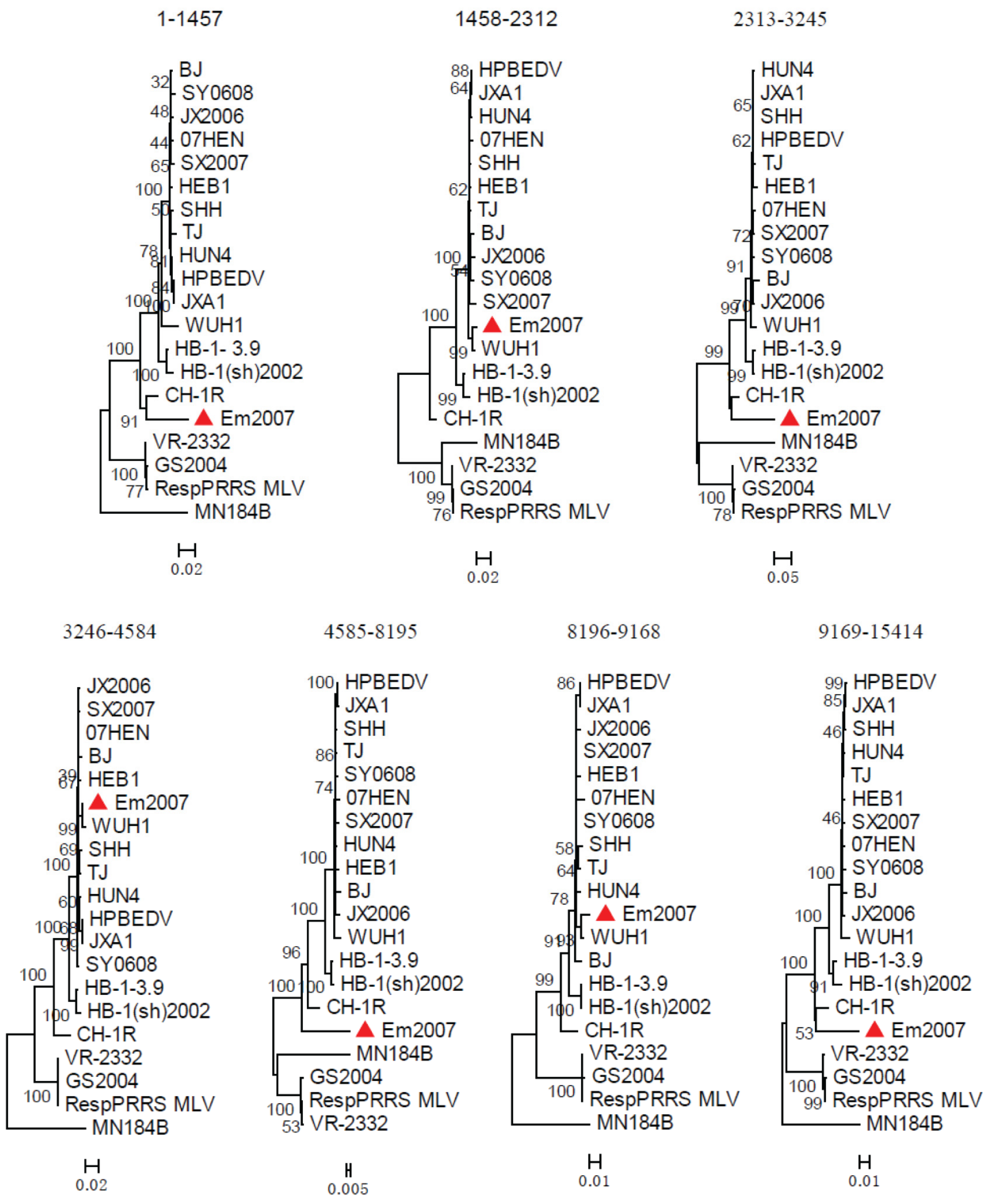

Figure 2. Genetic characteristics of Em2007 strain of porcine reproductive and respiratory syndrome virus showing neighbor-jointing phylogenies inferred for the 7 regions delimited by the breakpoints. For clarity, only some representative strains were shown in the phylogenetic trees. The putative mosaic is indicated with ▲. Scale bars indicate nucleotide substitutions per site.

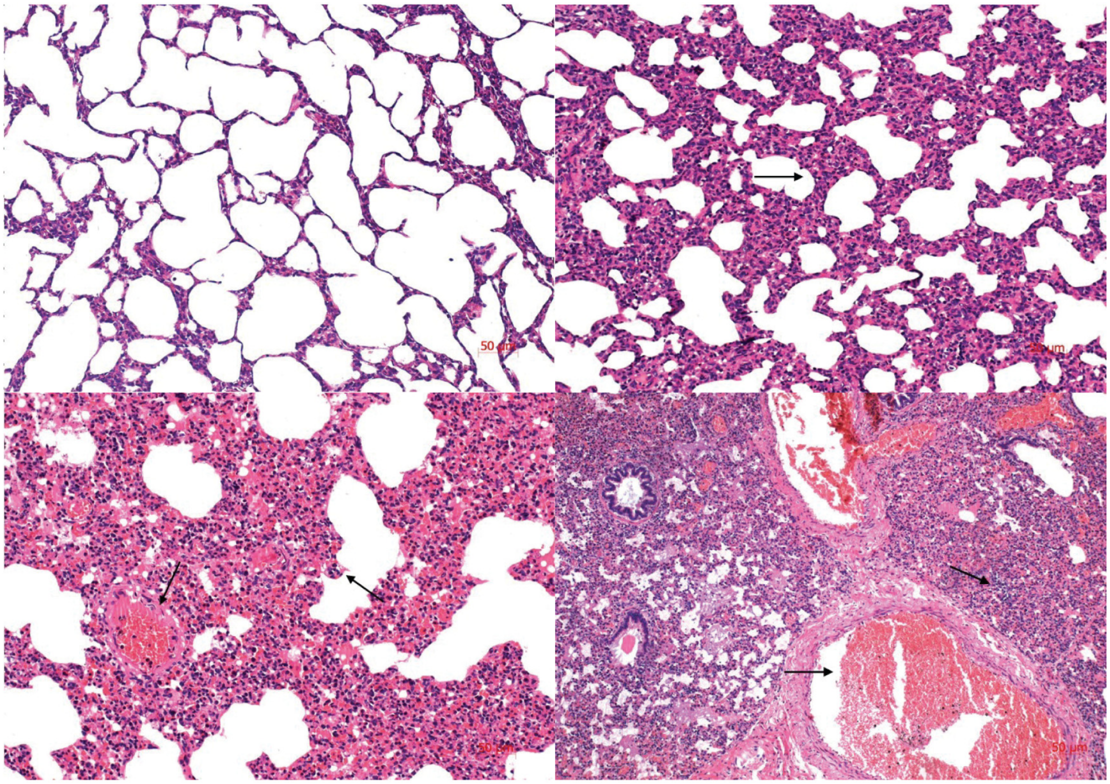

Figure 3. Histopathological comparison of hematoxylin and eosin-stained lungs at 10 days postinoculation with the Em2007, CH-1a and WUH1 strains of porcine reproductive and respiratory syndrome virus (PRRSV). Representative sections are shown. Control group (left, top panel), no lesions; CH-1a group (right, top panel), mild thickening (arrow) of the alveolar septa accompanied with infiltration of mononuclear cells; Em2007 group (left, bottom panel), marked thickening of alveolar septa by infiltration of mononuclear cells accompanied with hemorrhages slightly (arrow); WUH1 group (right, bottom panel), severe thickening of alveolar septa by infiltration of a large number of mononuclear cells accompanied with hemorrhages severely (arrow).
